# Heritability of cognitive performance in wild Western Australian magpies

**DOI:** 10.1098/rsos.231399

**Published:** 2024-03-13

**Authors:** Elizabeth M. Speechley, Benjamin J. Ashton, Alex Thornton, Leigh W. Simmons, Amanda R. Ridley

**Affiliations:** ^1^ Centre for Evolutionary Biology, School of Biological Sciences, University of Western Australia, Perth, Western Australia 6009, Australia; ^2^ School of Natural Sciences, Macquarie University, Sydney, New South Wales 2109, Australia; ^3^ Centre for Ecology and Conservation, University of Exeter, Penryn TR10 9FE, UK

**Keywords:** cognition, heritability, offspring, sociality, maternal effects, magpie

## Abstract

Individual differences in cognitive performance can have genetic, social and environmental components. Most research on the heritability of cognitive traits comes from humans or captive non-human animals, while less attention has been given to wild populations. Western Australian magpies (*Gymnorhina tibicen dorsalis,* hereafter magpies) show phenotypic variation in cognitive performance, which affects reproductive success. Despite high levels of individual repeatability, we do not know whether cognitive performance is heritable in this species. Here, we quantify the broad-sense heritability of associative learning ability in a wild population of Western Australian magpies. Specifically, we explore whether offspring associative learning performance is predicted by maternal associative learning performance or by the social environment (group size) when tested at three time points during the first year of life. We found little evidence that offspring associative learning performance is heritable, with an estimated broad-sense heritability of just −0.046 ± 0.084 (confidence interval: −0.234/0.140). However, complementing previous findings, we find that at 300 days post-fledging, individuals raised in larger groups passed the test in fewer trials compared with individuals from small groups. Our results highlight the pivotal influence of the social environment on cognitive development.

## 1. Introduction

Cognition is the mental process by which animals collect, retain and use information from their environment to guide their behaviour [1]. A substantial amount of research has investigated the factors underpinning the evolution of cognition [2]. Most studies have taken an interspecific approach to explore the ultimate mechanisms behind the evolution of cognition [3], but an increasing number of intraspecific studies have provided a detailed investigation of the factors influencing individual variation in cognitive performance [4,5]. Intraspecific variation in cognitive performance can be influenced by a variety of factors [6], including age [7], sex [8], personality [9] and the social environment [5]. However, a critical question in the evolution of cognition is the extent to which cognitive processes are adaptive and shaped by selection [10]. To be available for selection, cognition must show heritable variation [10]. Ultimately, quantifying the heritability of cognitive performance is a necessary step to understand the evolution of cognition [10].

Heritability, the proportion of variation in the phenotype that is attributable to genetic variance [11], provides a measure of the relative contributions of genetic and environmental factors. Most evidence of heritability in cognitive traits comes from humans [10], where studies have found that 30–50% of the variance in cognitive performance is genetic [12]. Studies examining the heritability of cognitive traits in non-human animals often do so in captive settings [13–16] that can be subject to inbreeding, founder effects and artificial selection [10]. As such, captive studies exploring the heritability of associative learning performance often report vastly different estimates [17,18]. For instance, a study on adult rose bitterling (*Rhodeus ocellatus*) found moderate heritability of learning accuracy *h* ^2^ = 0.27 [17], whereas a study on red jungle fowl chicks (*Gallus gallus*) found no evidence that associative learning was heritable (*h* ^2^ = 0.00) [18].

Genetic variance is not the only factor that can influence offspring phenotype: the environment during development can have a significant and lasting impact on an individual’s phenotype [19], particularly the social environment. In many species, parents play a major role in offspring development. Parental effects are a special case of intergenerational inheritance in which the environment provided by one or more parents affects the phenotype of offspring [20]. Maternal effects are traditionally defined as the influence of the maternally provided environment on offspring phenotype, and a source of variation that confounds the estimation of additive genetic variance [21]. More recently, maternal effects have been recognized as important factors in the evolution of life-history traits [22]. They describe the combined contributions of maternal genetic and environmental effects on offspring and provide estimates of the broad-sense heritability of traits (i.e. a combined measure of additive and epistatic genetic effects and maternal environmental effects). Relatively few studies explore maternal effects on cognitive performance [22,23], however those that do often find no relationship between mother and offspring cognitive abilities [23,24]. Additionally, the effects of interactions with other individuals (the social environment) on the development of cognition need to be considered. The genotype of social partner(s) may impact the focal individual’s phenotype via indirect genetic effects [25], and the social environment can play a pivotal role in shaping behaviour during developmentally sensitive periods. For example, Western Australian magpie fledglings (*Gymnorhina tibicen dorsalis*) raised in larger groups performed better in a cognitive test battery at 200 and 300 days post-fledging [26].

Here, we estimate broad-sense heritability (genetic and/or maternal effects) in magpies based on the regression of offspring on mother associative learning performance, while also investigating the potential impact of the social environment on cognitive development. Magpies present a model species to test these conditions because previous work has shown (i) phenotypic variation in cognitive performance in this species and (ii) that females with higher general cognitive performance have higher reproductive success [26]. Heritability is the last piece of the Darwinian puzzle for this species. Individual cognitive performance in this species is highly repeatable [27,28]. Given that repeatability can be used to set the upper limit to the heritability of a trait [11], the high repeatability in cognitive performance in magpies indicates that it may be heritable. As magpies have one of the highest rates of avian extra-group paternity on record (>80%) [29], without genomic data only the mother is reliably identifiable. Thus, although we are unable to estimate narrow-sense heritability due to fathers, we can estimate broad-sense heritability via mothers. In this study, we quantified associative learning in offspring at three time points during their first year of life to allow us to determine the factors affecting cognitive development. We predicted that genetic effects would appear early in development, whereas environmental effects would appear later [5].

## 2. Materials and methods

### 2.1. Study species

Magpies are a medium-sized (250–370 g) passerine living in cooperatively breeding groups varying between 3 and 12 adults [30,31]. Our study population was located in open grassland and parkland in Guildford (31°89′ S, 115°96′ E) and Crawley (31°98′ S, 115°81′ E), Western Australia [26]. The population has been monitored since 2014, and individuals are habituated to observers, allowing the presentation of cognitive tasks within 5 m [32]. Adult individuals in the population were individually identifiable through either coloured metal rings or plumage aberrations. During data collection, our study population consisted of 18 groups and 80–120 individuals.

The breeding season begins in August and lasts for several months [31]. All females in the group attempt to breed each season. Therefore, all nests were monitored daily during the breeding season to determine an accurate date of fledging for each individual. To avoid disturbance, nests were located visually using binoculars from a distance of >10 m and following mothers taking food to their offspring.

Magpie chicks spend approximately 3–4 weeks in the nest before fledging [31]. Fledging typically occurs from October to December each year. Once fledged, individuals remain in the natal territory and are reliant on group members for food until they begin to forage independently (~100 days post-fledging) [31]. However, fledglings continue to receive some care until they are approximately 200 days old [31].

### 2.2. Associative learning tests

We tested adult and fledgling cognitive performance in associative learning tests using methods similar to those employed by Ashton *et al.* [26]. Associative learning is a domain-general trait that is likely to be highly ecologically relevant because it allows animals to determine predictable associations between environmental cues, such as the behaviour of conspecifics [33,34]. Repeated testing is valid in this species, as previous research has shown that associative learning performance is highly repeatable [27], and cognitive performance does not improve with repeated testing via causally identical but visually distinct tasks [35] and does not differ between ringed and unringed individuals [36].

The associative learning array consisted of a wooden grid (31 cm × 9 cm × 4 cm) containing two equidistant wells (3.2 cm diameter × 1.5 cm depth, 3.5 cm apart). Wells were covered with PVC lids held in place by elastic bands threaded through drilled holes in the lids and fastened to the sides of the well, allowing the lids to swivel when pecked. PVC lids were painted with two different shades of a single colour, rather than two distinct colours, to limit pre-existing colour biases influencing performance [37,38]. Prior to testing, both wells were rubbed with cheese to reduce the possibility that olfactory cues could be used to locate the rewarded well. Prior to testing each individual, one shade (e.g. light green) was randomly selected to be the rewarded shade, meaning that the food reward would only ever be placed under that particular shade. For each trial, wells were baited out of sight from the individual, and the task was then placed 5 m in front of the individual. Once the task was placed down, the observer stepped back from the task (at least 5 m) to allow the individual to complete the array without interference. The subject was initially allowed to search both wells to see that only one well contained a reward, but in subsequent trials, they were only allowed to search one well before the apparatus was removed. The subject had a minimum 1-min interval between trials. An individual was considered to have passed the test when they selected the rewarded colour shade in 10 out of 12 consecutive trials (representing a significant deviation from random binomial probability) [26]. To ensure that a colour association was being measured (rather than a spatial association), the side of the rewarded location on the board was pseudo-randomized so that it was not on the same side for more than three consecutive trials. All individuals were tested in social isolation (with conspecifics at least 10 m away) to minimize the potentially confounding effect of social learning [26]. This was possible because magpies frequently forage 10–20 m away from each other [26].

**Figure 1.**
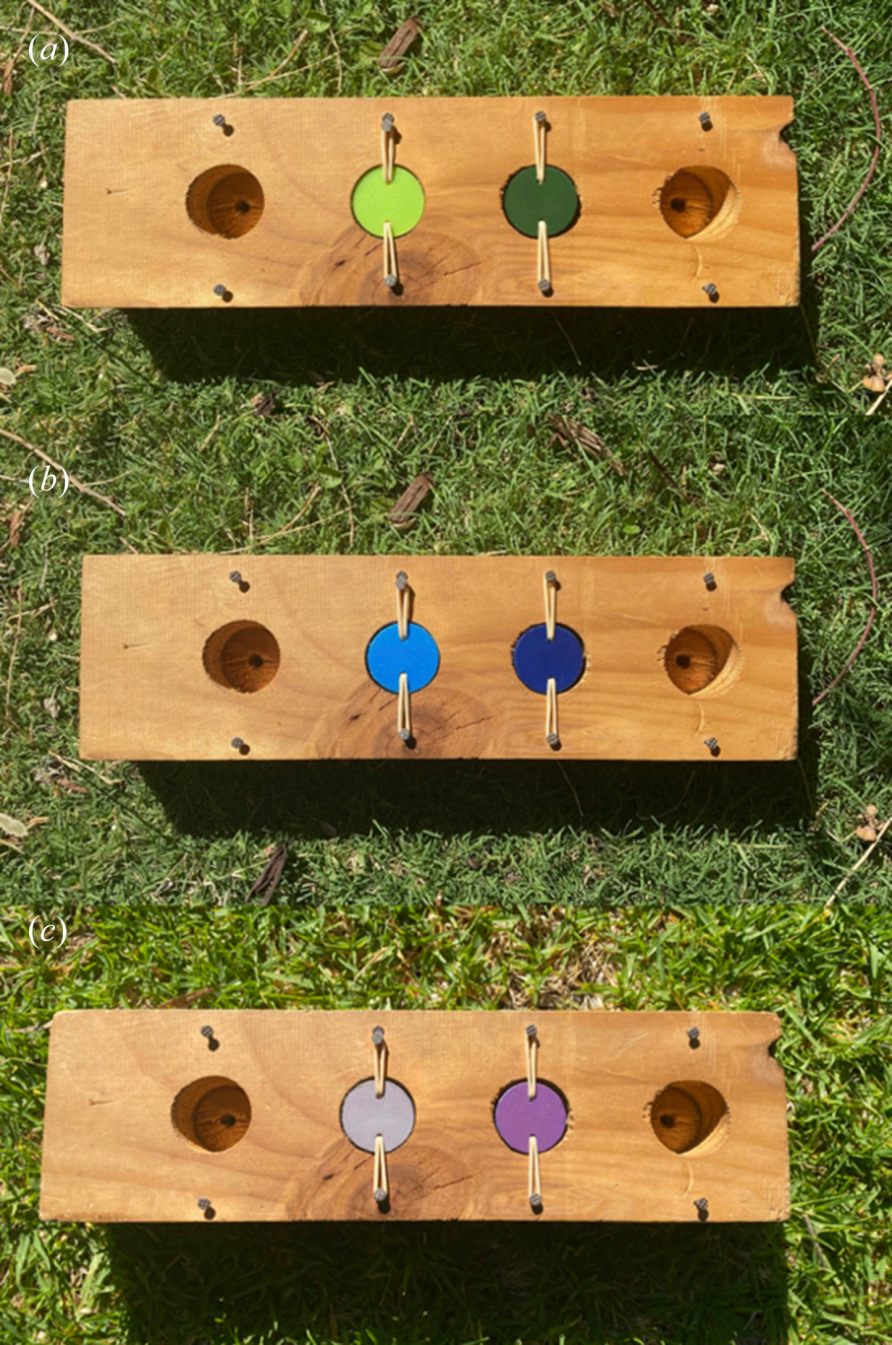
Associative learning array with colour combinations presented to fledglings at (*a*) 100, (*b*) 200 and (*c*) 300 days post-fledging. Each fledgling is randomly assigned a colour shade as the rewarded well at each testing period.

Fledglings were tested at three time periods during their first year of life: 100, 200 and 300 days post-fledging, following the developmental time periods identified by Ashton *et al.* [26]. Testing began at 100 days post-fledging because this is when individuals have started foraging independently and could readily engage with the cognitive task [26]. 200 days post-fledging corresponds to when most fledglings stop receiving care from other adults [31], and 300 days post-fledging corresponds to the period where fledglings transition to juveniles [26]. Tests for each testing period were causally identical but visually distinct: individuals were presented with a unique colour at each time period (figure 1). Cognitive testing was conducted in 2020 and 2021 during January–March (100 days post-fledging), April–June (200 days post-fledging) and August–September (300 days post-fledging) at randomized times from 5:00 to 11:00.

Maternal cognitive scores were obtained during March to April (during the non-breeding season) in 2020 or 2021, and where scores existed for both years, we used the score that was obtained closest to the date the fledgling was tested. To prevent any potential effects of prior learning on performance, individuals were always presented with a colour that they had not previously been exposed to (i.e. pink in 2020 and blue in 2021, apart from one female who was presented with purple in 2021; electronic supplementary material, figure S1).

### 2.3. Explanatory factors

We recorded several other factors that may influence cognitive performance and thereby confound the estimation of maternal effects, including the shade of the rewarded well (light or dark), neophobia (time taken to contact test apparatus from 1 m away), body mass (grams), foraging efficiency (amount of food caught in grams per minute foraging) and order tested. We also measured average ambient temperature at time of testing (°C), which is known to impact cognitive performance in this species [39].

We collected daily measurements of body mass by enticing individuals to hop onto a top pan scale (Ohaus Challenger series, 1000 ± 1 g) for a small food reward (<1 g mozzarella cheese) [30]. Foraging focal observations were recorded using a customized programme in Cybertracker [40] and individual foraging efficiency was calculated as the amount of food (in grams) caught per minute of time spent foraging [30]. Body mass and foraging focals were collected during the morning (5:00–11:00) within a week of cognitive testing. We also recorded latency to contact the task as a proxy of motivation and neophobia. Latency was defined as the time elapsed between approaching to within 1 m of the apparatus and first making contact with the apparatus.

### 2.4. Statistical analysis

All statistical analyses were conducted in R [41] (v.4.2.1). The data were checked to confirm they met model assumptions using the *DHARMa* package [42].

Prior to the main analysis, we ran a series of exploratory generalized linear mixed models (GLMMs) with a Poisson distribution using the *lme4* package [43] (separate GLMMs for each time period: 100, 200 and 300 days, *n* = 23, 24 and 17, respectively). These preliminary analyses were designed to test potential factors that might confound maternal effects on cognitive performance, including the shade of rewarded well, neophobia, body mass, foraging efficiency, average ambient temperature and order tested as explanatory terms. Offspring associative learning score (number of trials taken to pass the task) was the response term, and group identity was included as a random term.

To test the hypotheses that maternal score or the social environment affected fledgling associative learning score, we ran a series of GLMMs with a Poisson distribution on a subset of data where maternal associative learning score was available. Offspring associative learning score (number of trials taken to pass the task) was the response term. To explore the influence of the social environment, we included the total number of fledglings in the group and total group size as explanatory factors. The total number of fledglings was included because fledglings spend a substantial amount of time playing with other fledglings (personal observation) that may impact their cognitive development [44], whereas the total group size was included as a proxy for social interaction with all group members [31]. We also included maternal score as an explanatory factor to explore maternal effects. Group identity was included as a random term. We determined the best model using a model selection process, and terms were ranked in the order of their corrected Akaike Information Criterion (AICc) score (corrected for small sample sizes [45]). If a model was within two ΔAICc units of the best model (lowest AICc) and the effects had 95% confidence intervals (CIs) that did not intersect zero, it was included in the top model set. Sample sizes reflect the datasets after removing missing values.

To estimate the magnitude of broad-sense heritability, we calculated a mid-offspring value per female by averaging offspring scores where multiple scores were obtained from the same mother [11]. We also scaled the data around the mean and standard deviation for analysis [46]. We then estimated broad-sense heritability (genetic and/or maternal effects) by doubling the standardized regression coefficient of offspring on mother cognitive performance at each testing period as per the equation (*b* = 1/2 *h* ^2^, where *h* ^2^ represents heritability) for regression between offspring and one parent [11].

## 3. Results

We tested 23 individuals from 8 groups at 100 days post-fledging, 24 individuals from 8 groups at 200 days post-fledging and 17 individuals from 8 groups at 300 days post-fledging. The lower sample size at 300 days was a consequence of offspring mortality. Fifteen individuals completed the associative learning task at all three testing periods. The average number of fledglings per group was 2.11 ± 0.35. The subset of individuals with corresponding maternal scores was 19 individuals from 8 groups at 100 days post-fledging, 21 individuals from 8 groups at 200 days post-fledging and 15 individuals from 8 groups at 300 days post-fledging.

**Figure 2.**
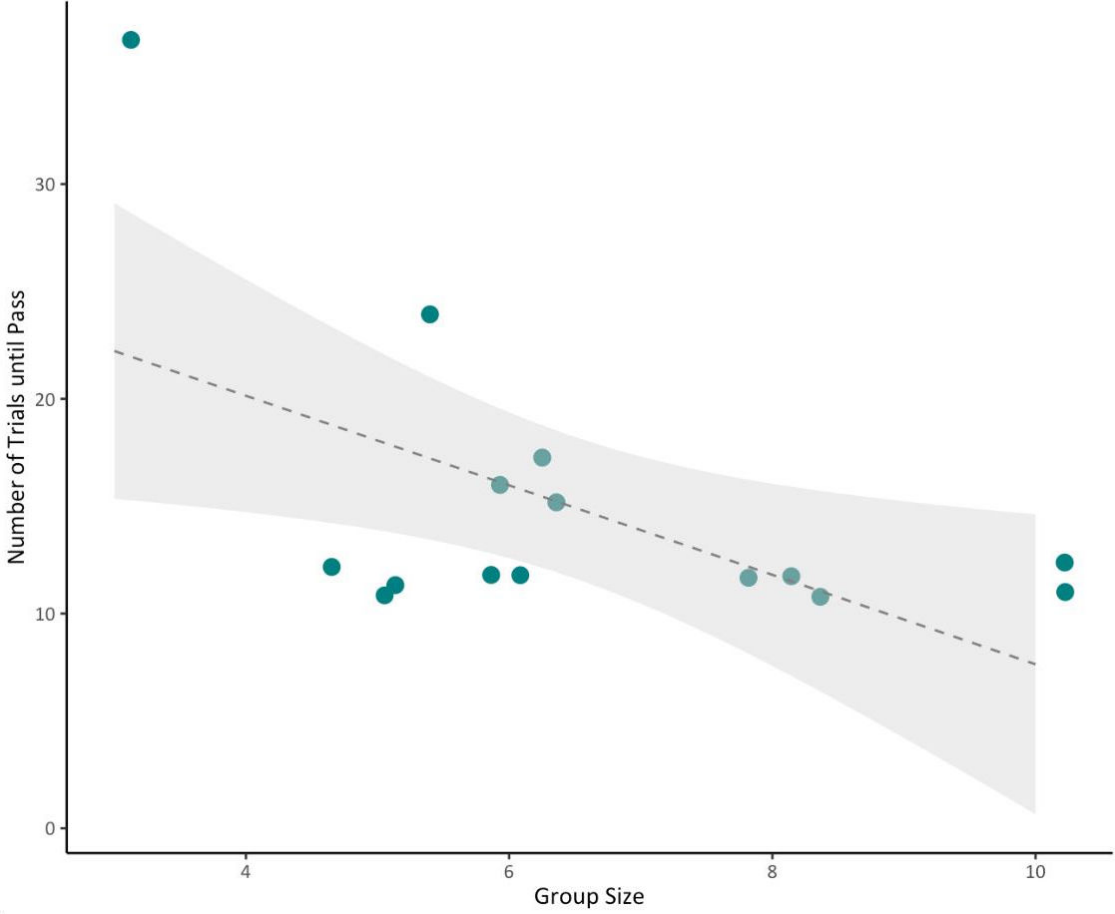
Relationship between group size and the number of trials taken to complete the associative learning test at 300 days post-fledging (*n* = 15 individuals from 8 groups). Points are raw data (jittered for visibility), fitted line represents the regression line and the shaded area represents the 95% CI.

Given the effect of shade identified in exploratory analyses for fledglings at 100 days (electronic supplementary material, table S1a), we included shade as an explanatory term in the main analysis for this developmental period. There was no effect of shade at 200 and 300 days post-fledging (electronic supplementary material, table S1b,c). At 100 days post-fledging, offspring score was predicted by shade of the rewarded well, with fledglings solving the task in fewer trials when the rewarded well was the lighter shade (estimate: −0.409, SE = 0.120, CI = −0.644/−0.175; electronic supplementary material, figure S2; electronic supplementary material, table S1a). None of our explanatory terms predicted cognitive performance at 200 days post-fledging (table 1b). At 300 days, the group size predicted offspring cognitive performance (estimate = −0.143, SE = 0.049, CI = −0.230/−0.050), where individuals raised in larger groups performed better in associative learning tests (table 1c, figure 2).

**Table 1.** Full model set of candidate terms affecting fledgling performance in the associative learning test using GLMM (Poisson distribution) at (a) 100 (*n* = 19), (b) 200 (*n* = 21) and (c) 300 days post-fledging (*n* = 15). Group identity was included as a random term. Corrected AICc and ΔAICc are provided for all models.

**Term**	**AICc**	**ΔAICc**
(a) 100 days		
colour shade	116.33	0
maternal score	121.59	5.26
group size	124.26	7.93
basic/null	124.62	8.29
number of fledglings	127.29	10.96
(b) 200 days		
basic/null	121.75	0
group size	122.36	0.61
maternal score	124.10	2.35
number of fledglings	124.49	2.74
(c) 300 days		
group size	93.74	0
basic/null	96.98	3.24
number of fledglings	98.59	4.85
maternal score	100.16	6.42

Based on the mother–offspring regression the broad-sense heritability estimate was −0.116 ± 0.076 (CI: −0.282/0.480) at 100 days (*n* = 14), 0.086 ± 0.078 (CI: −0.080/0.254) at 200 days (*n* = 15) and −0.046 ± 0.084 (CI: −0.234/0.140) at 300 days (*n* = 12).

## 4. Discussion

We aimed to estimate broad-sense heritability (genetic and/or maternal effects) in magpies based on the regression of offspring on mother-associative learning performance, while also investigating the potential impact of the social environment on cognitive development. Given previously high within-individual repeatability of cognitive performance, we predicted that cognitive performance would be heritable. However, our measure of broad-sense heritability provides little evidence for genetic and/or maternal effects on offspring cognitive performance. Our results instead complement previous findings on this species [26], indicating that individual variation in cognitive performance is underpinned by social factors during development, with offspring cognitive performance predicted by the group size at 300 days post-fledging.

We detected an effect of shade on associative learning performance at 100 days (but not the other two developmental stages), whereby individuals assigned the light green shade performed better. It is possible that there is a pre-existing bias toward the light green shade that is influencing performance. Other studies have detected a colour bias in associative learning tests; however, these usually involve a choice between two distinct colours (e.g. red versus blue) [38,47,48]. A shade bias in our study seems unlikely given that previous work on fledglings, and adults using this exact colour combination have not found a bias toward a particular shade nor has any other colour or shade bias been detected in fledglings or adults [26]. Therefore, it seems more probable that this result represents an inadvertent outcome of sampling (individuals with higher cognitive performance were by chance allocated the lighter shade as the rewarded well for their 100-day tests) rather than a true shade preference.

Our results reinforce the pivotal role that the social environment plays in the development of offspring cognitive performance in magpies. Specifically, we find a relationship between the offspring associative learning score and the group size, where offspring raised in larger groups perform better in cognitive tasks at 300 days post-fledging. Our results complement previous research on this population that also detected a relationship between the rearing group size and general cognitive performance in fledglings [26]. Similar results have also been reported in other species. For instance, cichlids (*Neolamprologus pulcher*) raised in larger social groups showed superior social competence [49]. Our results support the idea that living in large social groups may impose cognitive demands [50–52] and that these can shape cognitive development. Since magpies live in dynamic social groups, fledglings interact with many more group members than just their parents (via affiliative, agonistic and vocal interactions), which may help to explain the cognition–group size relationship. Additionally, magpies are cooperative breeders, with helpers consisting of adults and juveniles of both sexes [31] that results in fledglings interacting with many social group members.

Our estimate of broad-sense heritability suggests little, if any, heritability of associative learning performance in this species. Furthermore, the regression of mother and offspring scores provides no evidence of maternal effects on cognition. This suggests that cognitive performance is not determined by the maternal genotype or pre- or post-natal conditions provided by the mother. Several previous studies report low to moderate heritability of associative learning performance [14,17,53]. However, others, such as a study on red jungle fowl (*Gallus gallus*) [18] and wild adult North Island robins (*Petroica longipes*) [54], found no evidence of heritability for associative learning. We acknowledge that a small sample size and our ability to identify only mothers may limit our power to detect heritability. Therefore, we would encourage future studies to use genetic analysis based on blood samples collected during ringing to determine the relatedness of offspring to mothers and fathers and allow the calculation of narrow-sense heritability using a more powerful pedigree design. Additionally, we would encourage extending this analysis to other cognitive traits to gain greater insight into the heritability of cognitive performance in this species.

Our results suggest that cognitive performance may not be under selection in this species or that heritability may be low because genetic variation has been lost via selection. However, evolution can proceed due to environmental conditions, even in the absence of additive genetic variance in the relevant traits [55]. Recent research has adopted an approach that considers genes expressed in social partners (indirect genetic effects) as part of the environmental context that shapes animal behaviour [26,55]. When there is a social environment, the genetic differences between interacting individuals can contribute to differences in the social environment, creating a heritable environmental effect. Since magpies live in social groups and regularly interact with a range of different individuals [31], it is perhaps unsurprising to find offspring cognitive performance is influenced by social interactions beyond the mother. Therefore, cognition may be an emergent property of heritable variation in a host of different traits that are under selection through the social environment. Consequently, the evolution of cognition may be better modelled in the context of indirect genetic effects that capture the indirect influence on phenotypes. Future studies could explore this by monitoring the help fledglings receive from group members as per Pike *et al*. [31] and comparing the cognitive scores of these helpers to the cognitive development of fledglings.

Overall, we find that offspring cognitive performance is predicted by the social environment, but there is little evidence to suggest that there is broad-sense heritability of cognition or maternal environmental effects. In combination, our results suggest that the social environment may play a more important role in cognitive development than maternal cognition, and the evolution of cognition might be better modelled in the context of indirect genetic effects.

## Data Availability

Raw data, R code can be accessed via Dryad Digital Repository [56].
